# The Unusual Mass of Retrovesical Space: A Secondary Hydatid Cyst D?sease

**DOI:** 10.1100/tsw.2006.386

**Published:** 2006-10-02

**Authors:** Can Tuygun, Hasan Bakirtas, M. Abdurrahim Imamoglu, Nurettin Sertcelik, Kursad Zengin, Ibrahim Halil Bozkurt

**Affiliations:** Department of Urology, S.B. Diskapi Yildirim Beyazit Training and Research Hospital, Ankara, Turkey

**Keywords:** hydatid cyst, seminal vesicles, capitonnage, partial cystectomy

## Abstract

Hydatic cyst of seminal vesicles is very rarely seen. We report a case who complained of the inability to void, which developed progressively with dysuria, frequency, nocturia, and tenesmus, due to a giant retrovesical hydatid cyst that displaced the bladder and rectosigmoid region.

## CASE REPORT

A 62-year-old man applied to our clinic with voiding obstruction complaints and tenesmus. We placed a urethral catheter and drained about 800 cc residue urine. On bimanual rectal examination, we palpated a soft mass of which contours were not clear. On intravenous urography (IVU), we saw multiple calcified lesions, functional kidneys, impression sign below the bladder, and too much residue urine (Fig. 1). On abdominal tomography, many calcified septated cystic lesions in different sizes were seen in the liver, spleen, and left mesenteric lipoid tissue. There was also a septated cystic lesion with calcified walls measuring 90 × 80 mm in size on the posterior part of the bladder (Fig. 2). To better understand the type and nature of the lesion, we performed magnetic resonance imaging, and observed septated, thin-walled, not-pathologically-contrasted type 3 hydatid cysts, 88 × 98 × 110 mm in size, pushing the bladder to anterior and superior direction. Indirect hemagglutination test was positive to a dilution of 1/160. The patient described no hemospermia or other ejaculation problems associated with seminal vesical involvement of the hydatid cyst. Albendazole regimen was initiated 3 days before and continued after surgery for 1 month to limit the implantation of any unrecognized spillage during surgery.

The suprapubic incision and intraperitoneal approach was preferred to remove the retrovesical cyst. The tissue around the cyst was covered with sponges soaked with 3% saline solution to prevent intraperitoneal dissemination. During the dissection, the cyst was seen to originate from bilateral vesicula seminalis and both ductus deferens entered into the cyst (Fig. 3).

First, we placed two suspension sutures at the superior part of the cyst and checked the cyst content by a syringe. After confirming cyst hydatid, 3% hypertonic saline solution was injected into the cyst. Injected solution was kept in the cyst about 10 min and was then withdrawn. We made a small incision at the cyst wall for aspiration (Fig. 4). After this procedure, the germinative membranes removed by the aspirator and internal wall of cyst were cleaned with sponges soaked with 3% hypertonic saline solution.

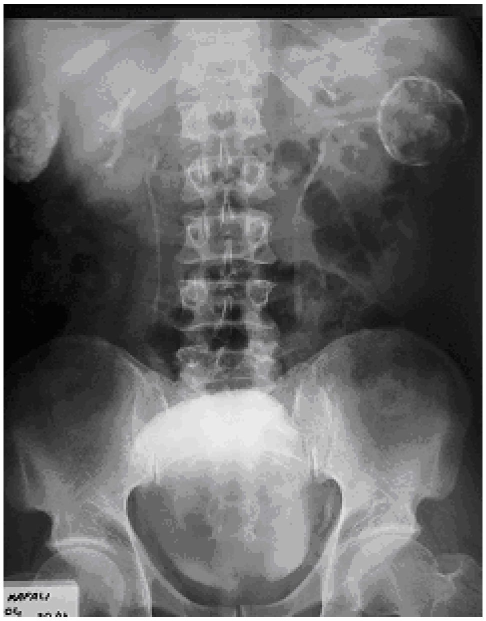

**FIGURE 1**. Multiple calcified lesions and functional kidneys on IVU.

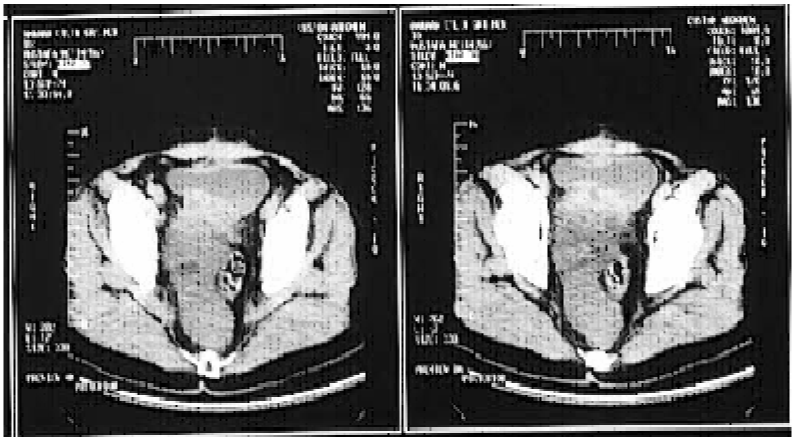

**FIGURE 2**. Localization of retrovesical mass and impression of it to adjacent organs on CT.

We performed a partial cystectomy and capitonnage procedure since the wall of the cystic mass was very adherent to the rectosigmoid and pararectal region, making proper dissection impossible. Surgical specimen including cyst wall, bilateral ducts, daughter vesicles, and germinative membranes is shown in Fig. 5. No postoperative complication was observed and the patient was discharged from hospital on the postoperative 7th day. At postoperative 6th month, control indirect hemagglutination test was found positive to a dilution of 1/320, because of other hydatid cyst lesions in the abdomen, but the patient had no voiding complaints. A hypodens lesion about 30 × 25 mm in size on the right posterior border of the bladder was seen in the control CT imaging of the abdomen (Fig. 6). When this hypodens lesion was checked by pelvic ultrasonography, the lesion was defined as cyst wall with no cystic formation, so the appearance was not correlated with recurrent hydatid cyst.

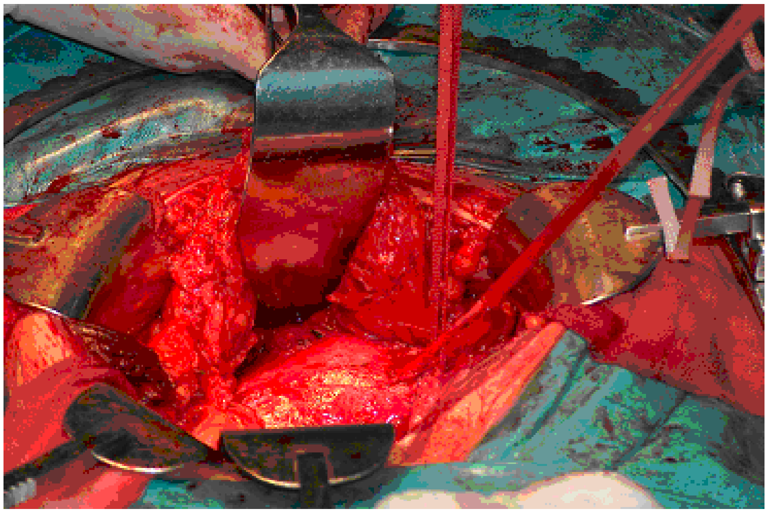

**FIGURE 3**. Eleveated right ductus deferens above cyst.

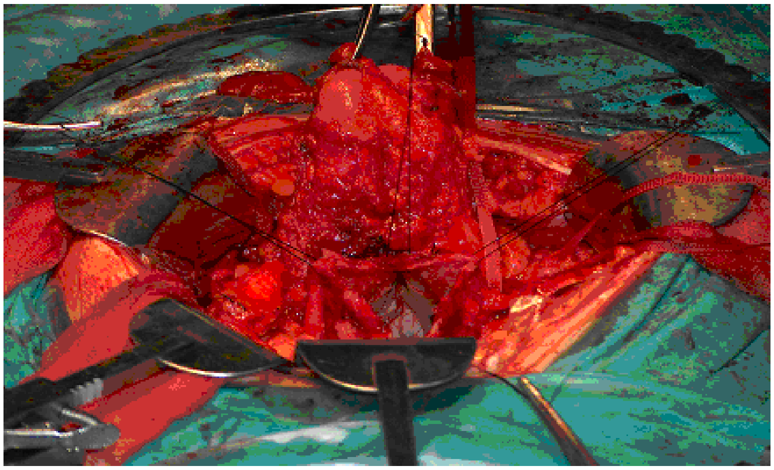

**FIGURE 4**. The view of opened cyst after sterilization.

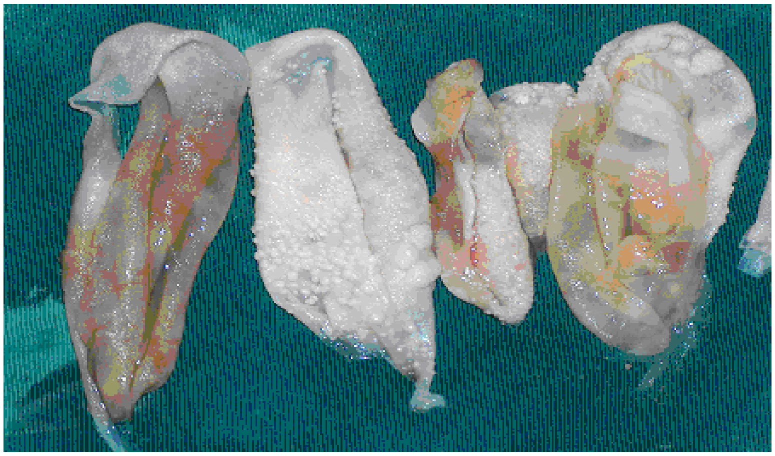

**FIGURE 5.** Germinative membranes belong to hydatid cyst.

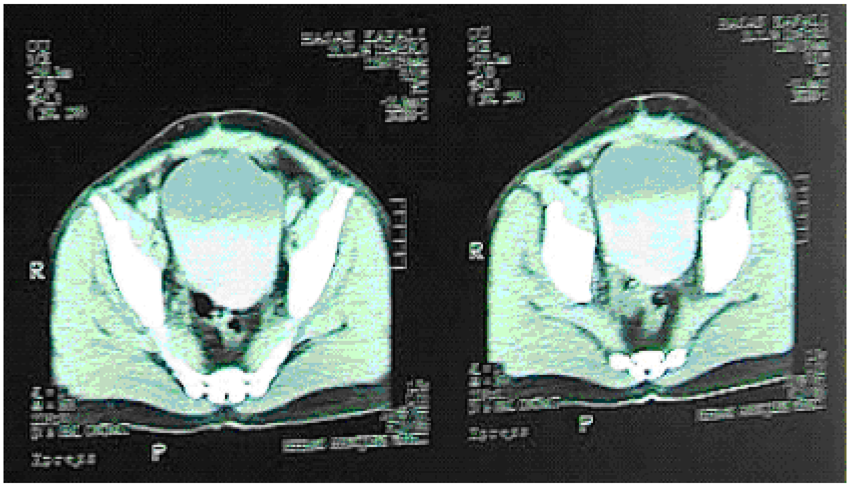

**FIGURE 6**. Hypodens lesion seen on right posterior side of bladder in postoperative 6th month on CT.

## DISCUSSION

The most common agent among *Echinococcus* genus is *E. granulosus,* and it is the most widespread in the world. It is especially seen endemically in Mediterranean countries, South America, Australia, New Zealand, and Middle East countries. It is one of the most prevalent parasites that cause hydatid disease[1,2,3]. In the urogenital system, hydatid cyst disease is a rare condition, especially at the rectovesical area. However, most cases are reported from our country, showing Turkey is an endemic region for hydatid cyst disease[1,2,3,4,5]**.** The retrovesical region is one of the primary sites of this disease[4,5,6], but it is mostly seen as secondary to the other organ involvement[3,7], either after surgery of the affected abdominal organ or by contamination from spontaneous or traumatic rupture of these. Patients may stay asymptomatic on rupture of the cyst, which may be diagnosed many years later while the patient is evaluated for other symptoms[3]. In our case, retrovesical involvement was thought to be secondary to spontaneous or traumatic rupture of other organs cysts.

The main therapeutic approaches for retrovesical hydatid cysts are total cystectomy or pericystectomy after aspiration and sterilization of the cyst cavity with scolecoidal solutions, such as hypertonic saline (3%), hydrogen peroxide, and 10% formalin[8]. The perforation of the cyst and adjacent organ injury due to aggressive dissection are the two most common major complications important in morbidity and mortality of the disease. It has been reported that pericystectomy is a more-preferred technique than total cystectomy in patients with very adherent cyst wall to adjacent organs[3,8]. We did not attend to remove the cyst totally due to severe adherence of the cyst to adjacent organs.

As a result, we think that a partial cystectomy and capitonnage method after emptying cyst content and sterilization is effective in patients who have adherent cyst wall to adjacent organs. Although aggressive procedures lower the risk of relapses, they increase the risk of complications. Therefore, surgical treatments of these patients must be individualized according to the severity of disease.
